# Socio-economic inequalities in the association between diabetes and labour force participation in Germany: A repeated cross-sectional study

**DOI:** 10.3205/000340

**Published:** 2025-06-30

**Authors:** Malwina M. Mackowiak, Ralph Brinks, Annika Hoyer, Ute Linnenkamp, Katharina Piedboeuf-Potyka, Markus Neuhäuser, Oliver Kuss, Thaddäus Tönnies

**Affiliations:** 1 Koblenz University of Applied Sciences, RheinAhrCampus, Department of Mathematics and Technology, Remagen, Germany; 2 Chair for Medical Biometry and Epidemiology, Witten/Herdecke University, Faculty of Health/School of Medicine, Witten, Germany; 3 Bielefeld University, Medical School OWL, Biostatistics and Medical Biometry, Bielefeld, Germany; 4 Institute for Health Services Research and Health Economics, German Diabetes Center (DDZ), Leibniz Center for Diabetes Research at Heinrich Heine University, Düsseldorf, Germany; 5 Institute for Biometrics and Epidemiology, German Diabetes Center (DDZ), Leibniz Center for Diabetes Research at Heinrich Heine University, Düsseldorf, Germany; 6 German Center for Diabetes Research, Partner Düsseldorf, München-Neuherberg, Germany; 7 Heinrich-Heine-University, Medical Faculty and University Hospital, Centre for Health and Society, Düsseldorf, Germany

**Keywords:** diabetes, labour force participation, socio-economic position, logistic regression, relative shortfall

## Abstract

**Objective:**

Diabetes is associated with lower labour force participation. The proportion of people having diabetes is higher among people with a low socio-economic position. We aimed to describe socio-economic differences in the association between diabetes and labour force participation in Germany.

**Methods:**

Based on repeated cross-sectional data from the German Socio-Economic Panel Study, the probability for participating in labour force was modelled with a logistic regression model including diabetes status, sex, socio-economic position, survey year and age as independent variables. Analyses accounted for the complex survey design of the study and used post-stratification weights. For easier interpretation, we estimated relative risks instead of odds ratios from logistic regression using post-estimation techniques. Relative labour force participation shortfall [%] was calculated as (1 – relative risk) x 100.

**Results:**

Labour force participation among people without diabetes was 82.2% compared to 55.9% among people with diabetes. Labour force participation shortfall was higher for low socio-economic position values and decreased with increasing socio-economic position. Labour force participation shortfall was generally larger among women while the association between labour force participation shortfall and socio-economic position was stronger among men.

**Conclusions:**

Diabetes-associated labour force participation shortfall mainly affects people with low socio-economic position, which indicates that this population subgroup not only carries a higher risk of diabetes, but also might be more strongly affected by its negative impact on productivity. Future studies aiming to quantify diabetes-associated productivity losses should take associations specific to socio-economic position into account.

## Zusammenfassung

**Hintergrund:**

Diabetes wird mit einer verminderten Erwerbsbeteiligung in Verbindung gebracht. Der Anteil an Personen mit Diabetes ist höher in der Bevölkerungsgruppe mit niedriger sozioökonomischer Position. Unser Ziel war es, Unterschiede in der Assoziation zwischen Diabetes und Erwerbsbeteiligung in Abhängigkeit von der sozioökonomischen Position in Deutschland zu beschreiben.

**Material und Methoden:**

Die Daten des sozioökonomischen Panels wurden als Querschnittsstudie mit wiederholten Messungen ausgewertet. Dabei wurde die Wahrscheinlichkeit einer Erwerbsbeteiligung unter Verwendung der Variablen Diabetesstatus, Geschlecht, sozioökonomische Position, Befragungsjahr und Alter als unabhängige Variablen mit Hilfe eines logistischen Modells analysiert. Das Befragungsdesign wurde innerhalb der Analyse berücksichtigt. Außerdem wurden Gewichte zur Post-Stratifizierung verwendet. Basierend auf den Ergebnissen aus der logistischen Regression haben wir relative Risiken ermittelt. Der relative Abfall in der Erwerbsbeteiligung [%] wurde durch (1 – relatives Risiko) x 100 berechnet.

**Ergebnisse:**

Die Erwerbsbeteiligung von Personen ohne Diabetes lag bei 82,2%, bei Personen mit Diabetes bei 55,9%. Der Abfall in der Erwerbsbeteiligung war höher für Menschen mit niedriger sozioökonomischer Position und mit steigender sozioökonomischer Position weniger ausgeprägt. Der Abfall in der Erwerbsbeteiligung war höher bei Frauen, wohingegen die Abhängigkeit zwischen dem Diabetes-assoziierten Abfall in der Erwerbsbeteiligung und der sozioökonomischen Position bei Männern stärker ausgeprägt war.

**Schlussfolgerung:**

Die Assoziation zwischen Diabetes und Abfall in der Erwerbsbeteiligung ist hauptsächlich bei Personen mit niedriger sozioökonomischer Position zu beobachten. Folglich ist diese Bevölkerungsgruppe nicht nur von einem größeren Risiko an Diabetes zu erkranken betroffen, sondern auch von einem größeren Diabetes-assoziierten Abfall in der Produktivität. Zukünftige Studien, welche den Zusammenhang zwischen Diabetes und Produktivitätsverlusten untersuchen, sollten daher die sozioökonomische Position in ihre Analysen einbeziehen.

## 1 Introduction

Diabetes mellitus has a negative impact on productivity resulting in substantial economic burden at both individual and population level. For instance, Bommer et al. [1] estimated that the global economic burden related to diabetes was equal to 1.8% of the global gross domestic product in 2015. In high-income countries, 40% of the costs were due to morbidity-associated production or productivity losses [1]. Reduced labour force participation (LFP), premature withdrawal from labour market and early retirement due to diabetes are amongst the factors influencing the productivity burden [2]. The length and amount of income during working life are the main determinants of pension entitlement in Germany [3]. Hence, individuals with diabetes may be at increased risk of income poverty in old age, given the reduction in productivity during working age. At the population level, the projected future increase in the prevalence of diabetes together with its negative impact on labour force may lead to an increasing economic burden in Germany [4], [5], [6].

It is well known that people in lower socio-economic position (SEP), as defined by education, occupation and income, are more often affected by diabetes, globally [7] and in Germany [8], [9]. In their systematic review, Agardh et al. [7] showed that the overall relative risk (RR) of developing Type 2 diabetes is 1.41 (95% confidence interval (CI): 1.28–1.51), 1.31 (95% CI: 1.09–1.57) and 1.40 (95% CI: 1.04–1.88) when comparing people with a low and high education, occupation and income, respectively. However, there are only few studies that investigated whether diabetes-associated productivity losses are higher among people with lower SEP compared to people with higher SEP. This is an important issue because it would imply that people with low SEP are severely disadvantaged in the labour market given their increased risk of diabetes and higher productivity losses associated with diabetes. For instance, Bender et al. [10] found that the association between Type 2 diabetes and disability pension risk in Denmark is larger among people with low compared to high educational level. Similarly, another study from Denmark found that working years lost due to diabetes are higher among people with lower education [11]. Nevertheless, this issue is not well explored and represented in the literature. Furthermore, not only education, but also income and occupation are important dimensions of SEP. Hence, we aimed to estimate SEP-specific associations between diabetes and LFP by age and sex in Germany. For this purpose, we estimated the LFP-shortfall associated with diabetes considering education, income and occupation combined in an SEP index using a large dataset representative for the German population.

## 2 Methods

### 2.1 Study design and data source

To investigate the association between diabetes and LFP, we performed a repeated cross-sectional study for the years 2009 to 2019, based on data from the German Socio-Economic Panel Study (GSOEP). The GSOEP is a nationally representative longitudinal household survey mainly based on face-to-face interviews [12]. We included individuals between 20 and 69 years of age, assuming that this is the relevant working age range, living in private households. The first information on diabetes status collected in the GSOEP was from 2009 [13], the most recent available data set at the time of our analysis was from 2019. As questions on diabetes were answered biannually [13], the analyses included data from every second survey year between the years 2009 and 2019. Further details on the GSOEP can be found in Goebel et al. [12] and in the web-based documentation of the data [13]. This study was approved by the Ethics Committee of Heinrich Heine University Düsseldorf, Germany (reference 2022-2219).

### 2.2 Variables

We defined LFP in accordance with the definition of the International Labour Organization [14]. Individuals were included in the group of labour force participants if they had a paid job during the previous seven days or if they were unemployed, available and actively seeking for a job during the past four weeks at the time of the interview. Accordingly, the group of labour force non-participants consisted of people who, at the time of the interview, did not have a paid job during the previous seven days and either i) did not intend to obtain employment in the future and/or ii) could not start an acceptable position within the next two weeks while not having actively searched for work within the last four weeks. Diabetes was defined as absent or present based on the question “Has a doctor ever diagnosed you to have one or more of the following illnesses?”. The revised version of the socio-economic status index described in Lampert et al. [15] was used as an indicator for SEP. The socio-economic status index combines information on education, income and professional position into a number ranging between 3.0 and 21.0 with lower values indicating a lower SEP. The educational level contains information on the highest individual educational qualification, while the occupational level is based on the highest position within the household at the time of the interview. Income is represented through gross equivalised disposable income on the household level [15]. To report results from the regression model, we categorized SEP values into three subgroups in order to oppose differences in relative LFP-shortfall when SEP values differ considerably. We defined groups by quintiles of the SEP distribution in the data as suggested in Lampert et al. [15]. The upper limit of the subgroup “low” was defined by the first quintile of the data. The limits of the broader subgroup “middle” were obtained through the first and fourth quintile. Accordingly, all individuals having an SEP value equal to or above the fourth quintile belong to the subgroup “high”. 

### 2.3 Statistical analysis

The probability for participating in labour force was modelled with a logistic regression model including diabetes status, sex, SEP, survey year and age as independent variables. We included a three-way interaction between diabetes, sex and SEP to allow for sex and SEP-specific associations between diabetes and LFP. SEP was included with a linear term while age was modelled with a linear and quadratic term to allow for a non-linear association. The survey year was modelled as a categorical variable. Since our aim was to describe the association between diabetes and LFP in sociodemographic subgroups in Germany, we did not account for potential confounders, e.g. obesity, comorbidities etc., but included sex, age, SEP and survey year besides diabetes as independent variables to allow for subgroup-specific estimates. 

The GSOEP uses a two-stage stratified cluster sampling procedure to select survey participants. Two-stage stratified sampling means that strata are sampled in the first stage. In the second stage, clusters (primary sampling units, PSU) are sampled within strata using a random walk procedure [12]. In our statistical analyses, we accounted for this complex survey design by using general maximum pseudolikelihoods and Taylor series approximation for variance estimation, as implemented in the R package “survey” [16]. Although the data are potentially clustered at multiple levels (e.g. survey respondent, household, PSU), we defined the highest level of aggregation, the PSU, as the cluster for the cluster-robust standard errors, as suggested in Cameron and Miller [17]. To ensure representativeness of our data set, we used post-stratification survey weights. Information on strata, PSU und post-stratification weights was provided in the GSOEP [13]. Probabilities of LFP were obtained using average adjusted predicted values. For easier interpretation, we used post-estimation techniques to obtain relative risks (RRs) instead of odds ratios from the logistic regression model. Specifically, we estimated average marginal RRs with standard errors based on the delta method, as it is implemented in the R package “marginaleffects” [18], [19]. Relative LFP-shortfall [%] was calculated as (1 – RR) x 100. As an alternative, one could use log-binomial regression to estimate RRs directly. However, log-binomial regression that accounts for the complex survey design is not implemented in standard statistical software which is why we used post-estimation from logistic regression. Another alternative for direct RR estimation is Poisson regression. Predicted probabilities can exceed the interval [0,1] when using Poisson regression for a binary outcome. As this was the case for our data, we determined that logistic regression, where the probabilities are limited to [0,1], followed by a post-estimation is a more reliable approach for obtaining RRs in this study. We present results from regression models for the midpoints of the corresponding SEP-groups as defined by survey-weighted quintiles of the GSOEP data, i.e. 5.8, 12.3 and 18.5 for low, middle and high SEP. All analyses were conducted with R (The R Foundation for Statistical Computing) version 4.1.3. 

## 3 Results

In total 100,706 observations from 39,273 participants were used excluding 8,090 observations with missing information for LFP, diabetes status, age, sex, SEP or survey year. Survey-weighted descriptive statistics of the study population are reported in Table 1 (Tab. 1). 

Overall, there were 5,930 observations with and 94,776 observations without diabetes. The distribution of participants with diabetes was comparable between the survey years ranging between 14.4% (survey year 2013) and 19.0% (survey year 2017). Among people without diabetes, 82.2% participated in labour force. This proportion was considerably lower for people with diabetes (55.9%). In the total sample, LFP was 80.6%. The mean age (±SD) of the total sample was 45.7 (±13.7) years, with a higher value for participants with diabetes (56.9±9.9 years) compared to those without diabetes (45.0±13.6 years). The weighted average (±SD) for SEP is less in persons with diabetes (10.8±3.7) indicating a lower SEP compared to persons without diabetes (12.1±3.8).

Based on average marginal effects from logistic regression, LFP was 8.34 percentage points (pp) (95% CI: 5.43–11.25) and 10.84 pp (95% CI: 6.60–15.09) lower among men and women with diabetes than among men and women without diabetes.

**Figure 1:**
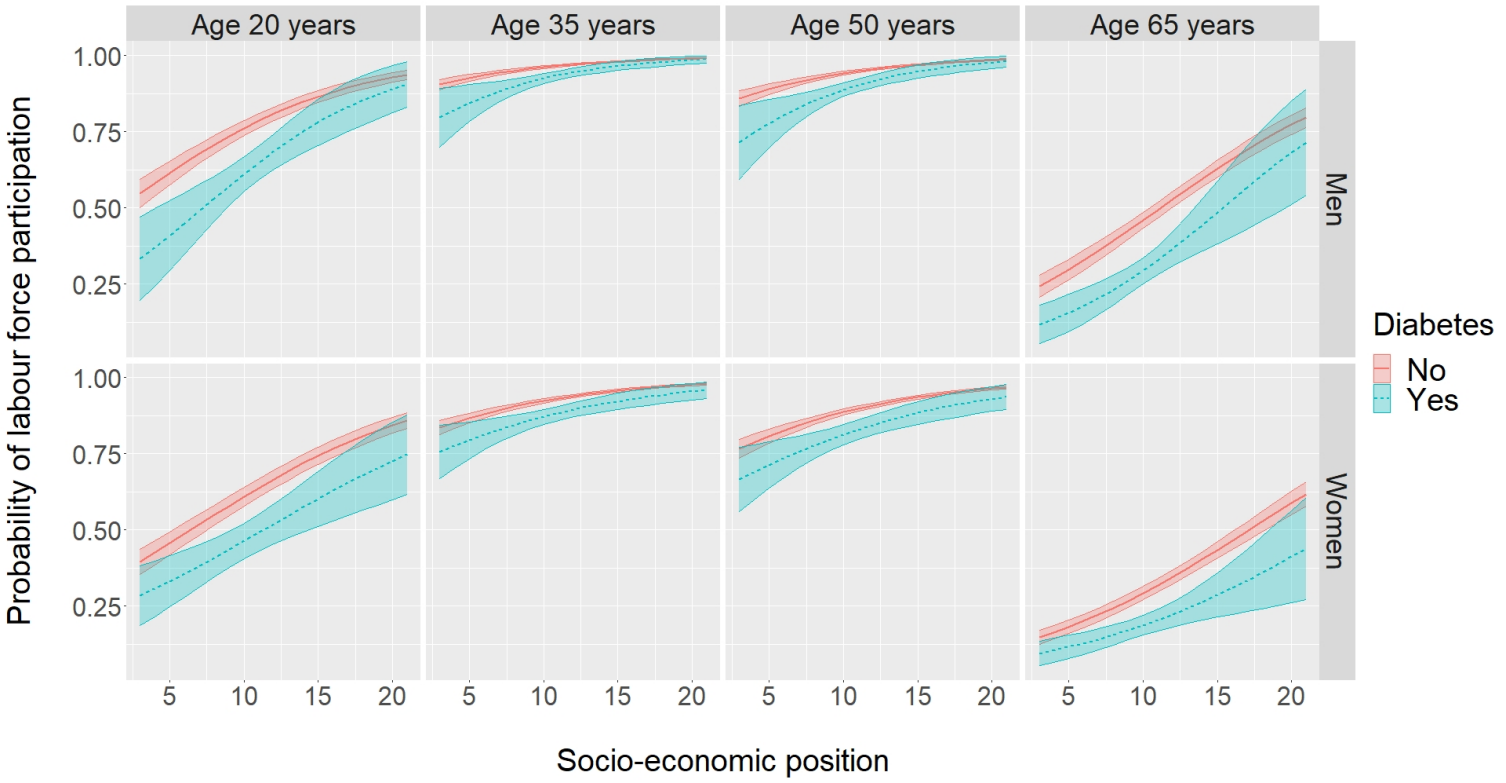
Probability of labour force participation for people with and without diabetes in dependence on socio-economic position (SEP), sex and age. Results are based on logistic regression including the following independent variables: a three-way interaction between diabetes, sex and a linear term for SEP, a linear and quadratic term for age and the survey year as a categorial variable. Predicted probabilities are presented for the survey year 2017.

Figure 1 (Fig. 1) shows predicted probabilities for LFP for people with and without diabetes by SEP, age and sex, based on logistic regression. Mean probabilities of LFP for men without diabetes were equal to 0.81 (95% CI: 0.79–0.84), 0.97 (95% CI: 0.97–0.97), 0.96 (95% CI: 0.95–0.96) and 0.54 (95% CI: 0.51–0.54) for ages 20, 35, 50 and 65 years and 0.70 (95% CI: 0.63–0.76), 0.95 (95% CI: 0.93–0.96), 0.92 (95% CI: 0.90–0.94) and 0.38 (95% CI: 0.32–0.44) for men with diabetes, respectively. The corresponding probabilities were 0.68 (95% CI: 0.65–0.70), 0.94 (95% CI: 0.94–0.95), 0.91 (95% CI: 0.90–0.92) and 0.36 (95% CI: 0.33–0.38) among women without diabetes and 0.53 (95% CI: 0.46–0.60), 0.90 (95% CI: 0.87–0.92), 0.85 (95% CI: 0.82–0.88) and 0.23 (95% CI: 0.19–0.27) among women with diabetes. Regarding the association with SEP, predicted LFP probabilities increased with an increasing SEP with a much steeper slope for age 20 and age 65 than for age 35 and age 50. Generally, LFP probabilities were lower in women than in men. The differences in LFP between people with and without diabetes were different for men and women. While differences decreased with an increasing SEP among men, this decrease was less pronounced among women aged 35 and 50. For women, SEP-specific differences were rather constant at age 20 years and increasing with higher SEP values at age 65 years. For men, this was somewhat inversed. Within each sex, LFP probability curve profiles were similar for individuals with and without diabetes at ages 35 and 50. 

**Figure 2:**
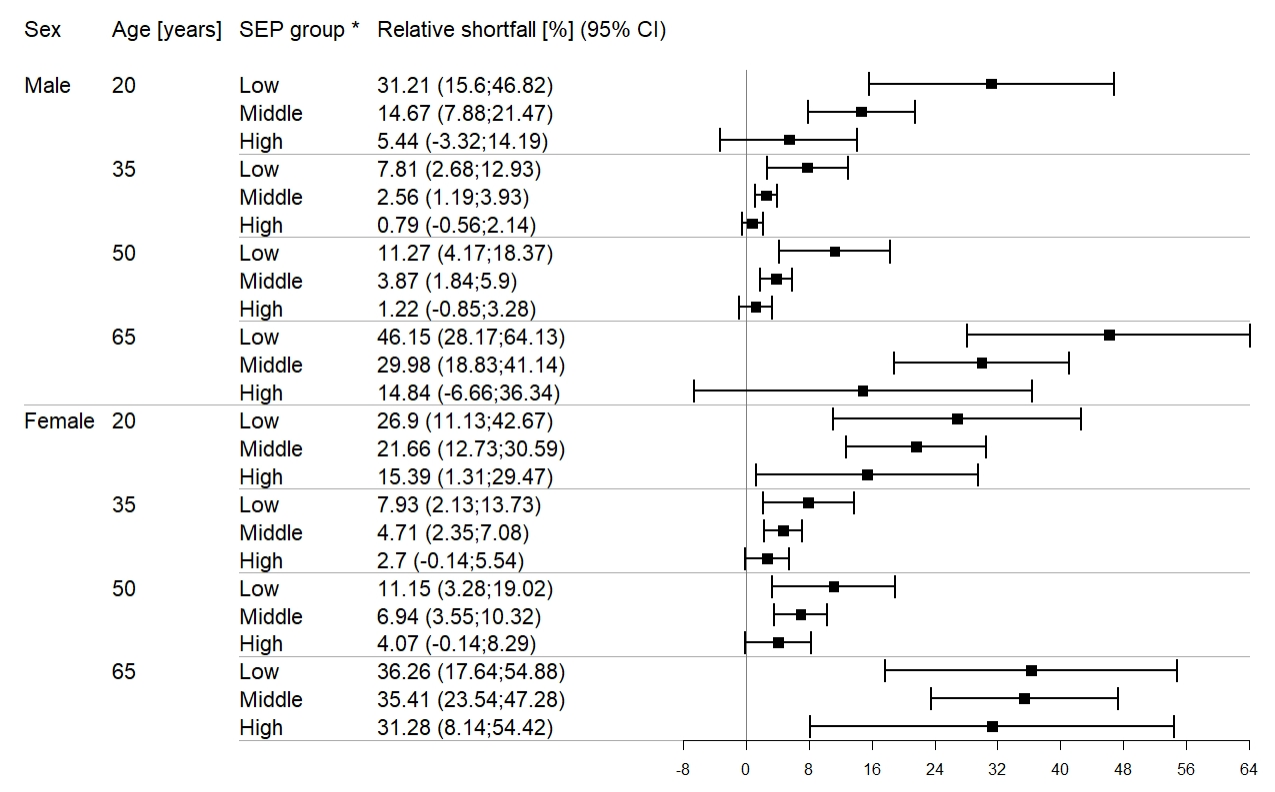
Relative labour force participation shortfall comparing people with diabetes to people without diabetes by sex, age and socio-economic position (SEP) group. Results are based on logistic regression including the following independent variables: a three-way interaction between diabetes, sex and a linear term for SEP, a linear and quadratic term for age and the survey year as a categorial variable. * Relative shortfall is presented for survey year 2017 using mid points between lower and upper bound of the respective SEP group, leading to 5.8 for “low”, 12.3 for “middle” and 18.5 for “high”.

Based on this predicted LFP, Figure 2 (Fig. 2) shows the relative LFP-shortfall associated with diabetes by sex, age and SEP group. The results in Figure 2 (Fig. 2) indicate that the LFP-shortfall was larger among women at all ages considered, except for the low SEP group. The largest difference in LFP-shortfall associated with diabetes was observed among men at age 65 years in the low SEP group (46.15%, 95% CI: 28.17%–64.13%). The sex difference in LFP-shortfall was largest at age 65 in the high SEP group (14.84% (95% CI: –6.66%–36.34%) for men vs. 31.28% (95% CI: 8.14%–54.42%) for women) and lowest at ages 35 and 50 in the low SEP group, respectively. When comparing the different age values, ages 35 and 50 show rather similar average adjusted relative shortfalls with a minimum of 0.79% and a maximum of 11.27%, while relative shortfalls are considerably larger for age 20 (minimum: 5.44%, maximum: 31.21%) and particularly high for age 65 (minimum: 14.84%, maximum: 46.15%). 

**Figure 3:**
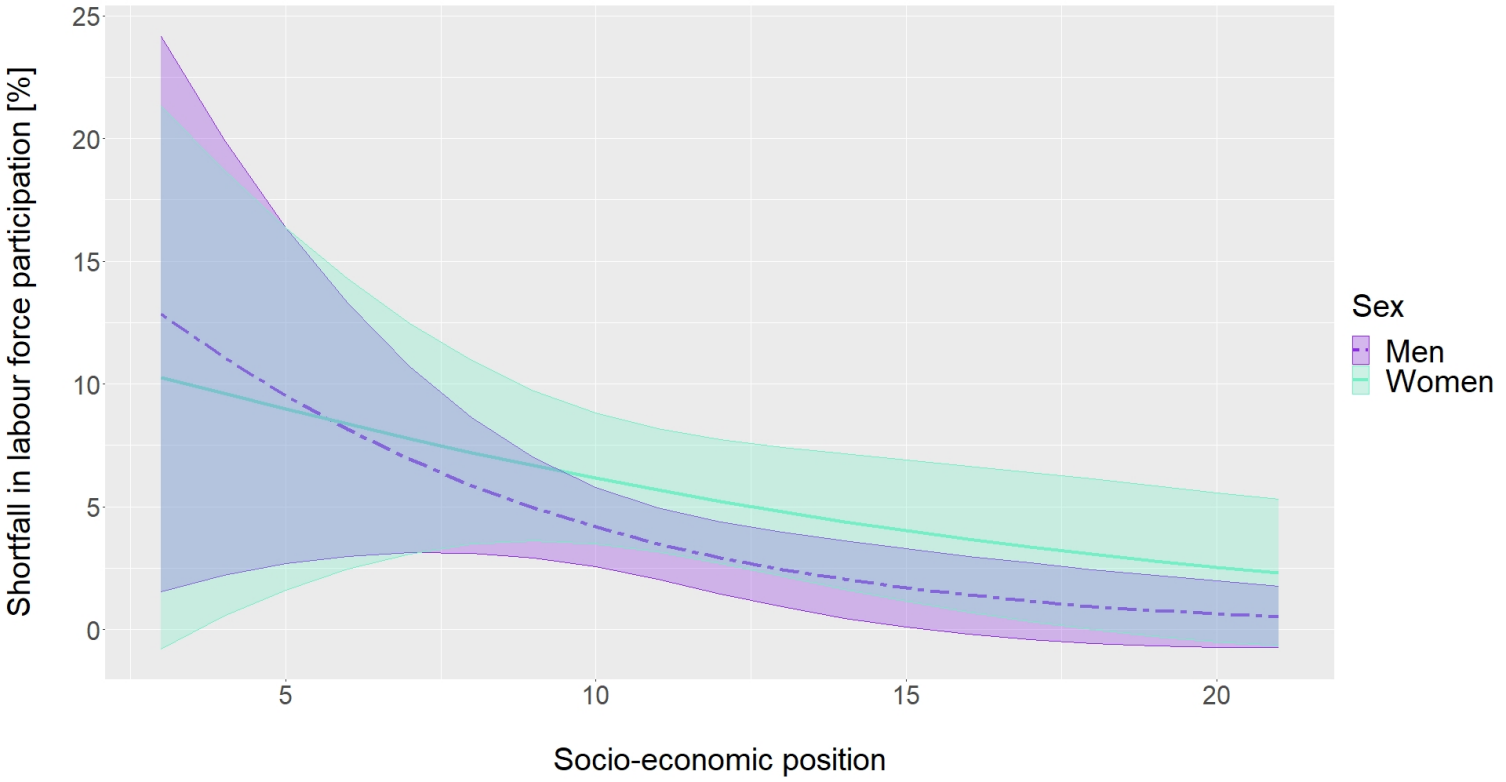
Labour force participation shortfall for people with diabetes compared to people without diabetes in dependence on socio-economic position (SEP) and sex. Results are based on logistic regression including the following independent variables: a three-way interaction between diabetes, sex and a linear term for SEP, a linear and quadratic term for age and the survey year as a categorial variable. Shortfall estimations are presented for the age of 46 years and the survey year 2017.

Figure 3 (Fig. 3) shows the LFP-shortfall in dependence on SEP and sex. In general, the relative LFP-shortfall was higher for low SEP values and decreased with increasing SEP. 

Model estimates from logistic regression can be found in Table 2 (Tab. 2). We added estimates from the corresponding model without interactions for comparison purposes. 

## 4 Discussion

### 4.1 Summary of findings

Our study, in which we examined the LFP-shortfall of people with diabetes relative to those without diabetes for different SEP groups, revealed that the relative LFP-shortfall was generally higher for low SEP and decreased with an increasing SEP. Thereby, the interaction between SEP and LFP-shortfall was slightly stronger among men.

Our findings agree very well with the literature in terms of the diabetes-associated loss in productivity. LFP was equal to 55.9% for individuals with diabetes in our study population, i.e. 26.3 pp lower than for individuals without diabetes. Therefore, a negative association between LFP and diabetes can be inferred as already stated in Köster et al. [20] and Tönnies et al. [5] by means of indirect costs and productivity-adjusted life years lost. 

Sex-specific assessments based on our model led to the conclusion that not only LFP was lower among women, which is in accordance with International Labour Organization [14] data, but the LFP-shortfall was also 2.5% greater in women when comparing persons with diabetes to those without diabetes. This outcome suggests that the negative impact associated with diabetes is generally stronger among women than among men in Germany. In their systematic review, Bommer et al. [1] found a LFP-shortfall of diabetes equal to 12.6% for men and 25.2% for women in high-income countries. Our model-based estimates were equal to 8.34% (95% CI: 5.43%–11.25%) for men and 10.84% (95% CI: 6.60%–15.09%) for women. Comparisons of LFP-shortfall by age revealed that estimates were higher for ages 35 and 50 years while being lower towards the beginning and end of working life, particularly for women, when opposed to our outcomes. These differences between global estimates from Bommer et al. [1] and our results suggest that country-specific estimates might be more useful to assess the productivity burden of diabetes.

LFP was less for ages 20 and 65 than for 35 and 50 years in our study. This is probably because those aged 20 were partly still in education and those aged 65 were mostly retired. In contrast, the LFP-shortfall associated with diabetes was largest for those aged 65, which is in line with Pedron et al. [2] who found a positive association between diabetes and the probability for early retirement. The estimates for age groups between 35 and 50 years, in which the social gradient seems to be less pronounced, might be more representative for the working population. To our knowledge, no previous study investigated SEP-specific associations between diabetes and LFP.

### 4.2 Implications for public health

People with a low SEP are more likely to have diabetes [7]. Additionally, our results indicate that diabetes-associated LFP-shortfall is more pronounced among individuals with a low SEP. Consequently, the productivity loss is particularly high in this population subgroup. Individuals do not only carry the burden of the disease, but also experience larger financial disadvantages than people with higher SEP and diabetes, leading to larger reductions in pension entitlements and potentially to income poverty in older age. To avoid this, efforts regarding diabetes prevention and treatment as well as improvement of socio-economic conditions should be intensified specifically for these individuals. For Germany, our findings suggest that labour force is lost due to diabetes, especially in the subgroup with a low SEP. Since the prevalence of diabetes is increasing [4], this circumstance results in a growing economic burden. Future investigations and measures to reduce the productivity burden associated with diabetes should therefore take into consideration that LFP and diabetes as well as LFP-shortfall associated with diabetes considerably depend on SEP. 

### 4.3 Strengths and limitations

A major strength of our study is the large sample size and the representativeness for the German population. The data contains detailed information on education, profession and income on individual and household levels, so that the SEP value could be derived as a summary of this information. Thereby, the use of SEP as a composite measure gives a more reliable indicator for the persons’ position compared to the use of education, income and profession alone since the SEP shows a higher correlation with the single components than the single components among each other [15]. As drawback, inferences on the single components of the composite measure cannot be made [21]. The incorporation of income on the household level for calculating individual SEP scores is more appropriate than using the individual income. This is because the income of other household members can directly affect the amount of disposable income of other household members. In turn, the same amount of income on the household level could result in different amounts of disposable income on the individual level, particularly when children are part of the household. However, there are potential sources of bias in our study. First, there may have been people with undiagnosed diabetes in the group of people without diabetes. These individuals are assumed to be rather asymptomatic and consequently are more likely to participate in labour force. As a consequence, LFP of people with diabetes would have been underestimated [2]. Second, the degree of misclassification could depend on SEP, for instance if people in lower SEP are more frequently affected by undiagnosed diabetes than people in higher SEP, which could introduce additional bias. The fact that information on diabetes was self-reported could be one further limitation since diagnoses based on laboratory measurements are more valid. However, a recent German cohort study in individuals aged 70–95 years showed that self-reported information on diabetes agrees very well with reports of general practitioners [22]. Also, the GSOEP does not differentiate between types of diabetes. It is assumed that at least 90% of all diabetes cases indicate cases of Type 2 diabetes [23]. The association of Type 2 diabetes on LFP is presumably larger than estimated in this study, since a non-distinction between diabetes types probably results in an underestimation of the true effect of Type 2 diabetes [2]. Studies included in the systematic review of Pedron et al. [2] showed that associations between Type 1 diabetes and employment are generally weaker than between Type 2 diabetes and employment. Therefore, estimations pooling data for both Type 1 and Type 2 diabetes may show a weaker negative association compared to estimations solely based on Type 2 diabetes. A further limitation is the potential for selection bias due to non-response. Although survey weighting addresses this issue to some degree, it does not guarantee representativeness. Given the considerable rate of non-response in the GSOEP (e.g. 23.4% in the year 2019), selection bias cannot be ruled out [24]. Another limitation is that our repeated cross-sectional study did not make use of the longitudinal information from survey respondents. We do not draw conclusions in terms of causal relationships and potential confounders, such as obesity, comorbidities etc. Our intention was to perform a descriptive analysis of the association between diabetes and LFP, as currently present in Germany, in order to assess the existence of inequalities, the need for action and support hypotheses generating processes and future research. In the future, measures taken to reduce these inequalities can be evaluated by comparing new data to our results. To investigate the causal relation between LFP, diabetes and SEP, the GSOEP data could be used by taking advantage of the longitudinal study design in a next step. One focus could be the bidirectional relationship between diabetes and SEP over time.

## 5 Conclusion

In summary, we found that diabetes-associated LFP-shortfall mainly affects people with low SEP, which indicates that this population subgroup not only carries the higher risk of diabetes, but also might be more strongly affected by its negative impact on productivity. Future studies quantifying the causal effect of diabetes on productivity losses should take SEP-specific effects into account.

### Key points

- The probability for participating in labour force is increasing with increasing socio-economic position.
- The labour force participation shortfall of people with diabetes relative to people without diabetes is substantially higher among persons with low socio-economic position.
- Efforts should be intensified to reduce diabetes incidence, particularly for people with low socio-economic position, in order to decrease economic burden due to productivity losses in Germany.

## Notes

### Acknowledgements

We thank the German Institute for Economic Research (DIW Berlin) for sharing data used in this publication and for their support.

### Data availability

The SOEP-Core data can be ordered for scientific purposes via the research data center of the SOEP at DIW Berlin. Details on the procedure can be found at https://www.diw.de/en/diw_01.c.601584.en/data_access.html.

### Funding

Funded by the Deutsche Forschungsgemeinschaft (DFG, German Research Foundation) – project number 516146656.

### Competing interests

The authors declare that they have no competing interests.

## Figures and Tables

**Table 1:**
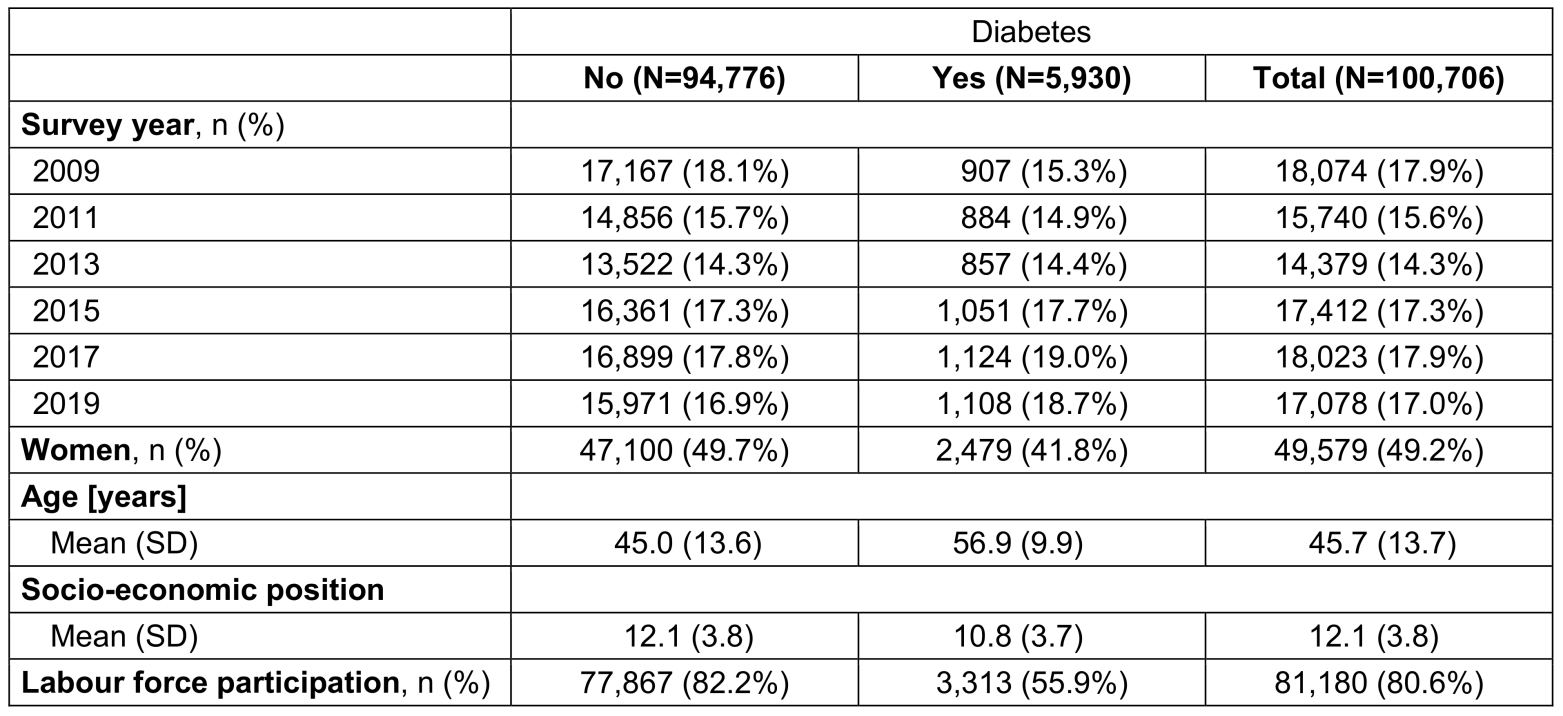
Survey-weighted characteristics of study population

**Table 2:**
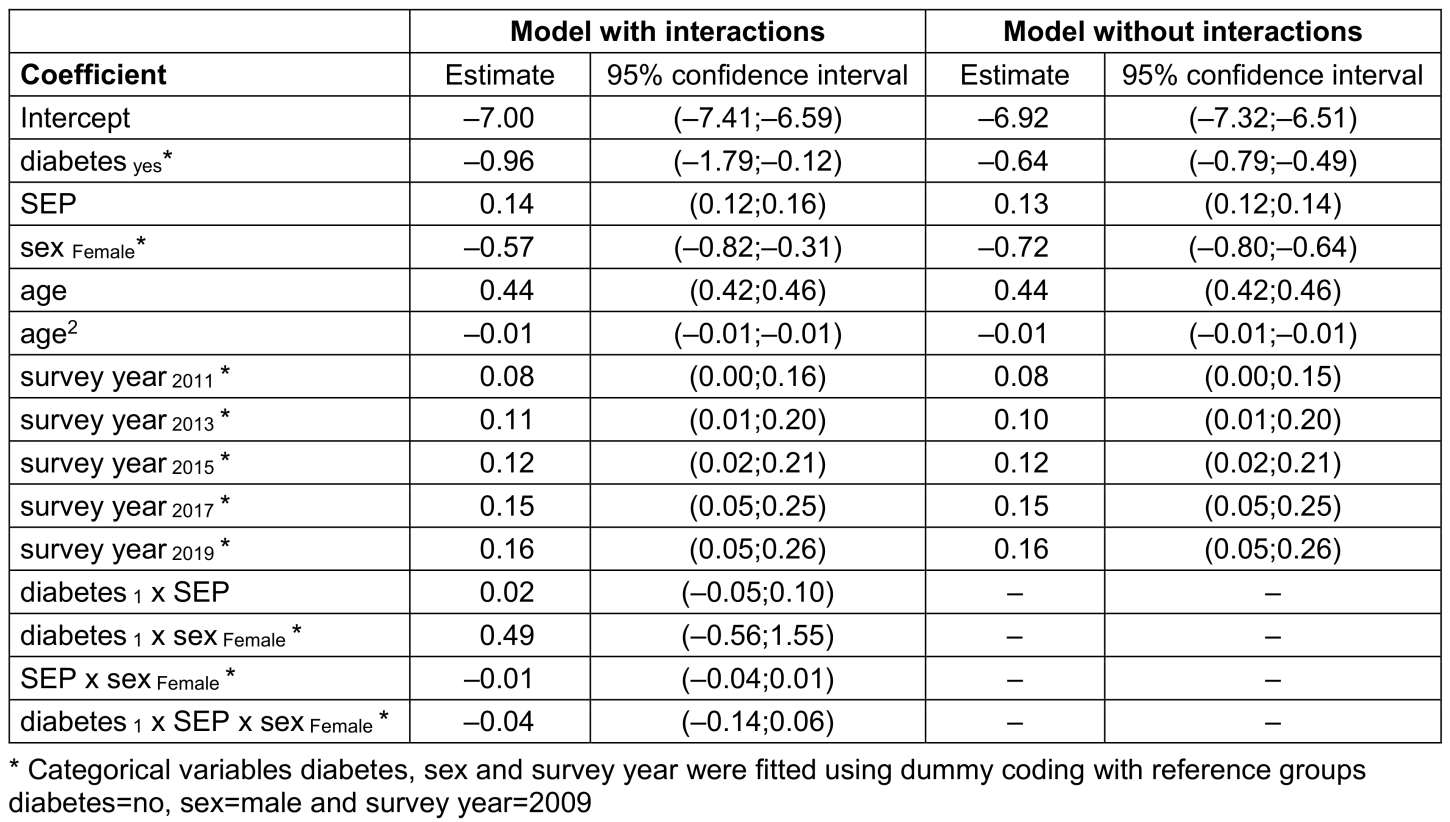
Parameter estimates and 95% confidence intervals from logistic regression models with and without interactions
